# Genomic Characterization and Safety Assessment of *Bifidobacterium breve* BS2-PB3 as Functional Food

**DOI:** 10.4014/jmb.2311.11031

**Published:** 2024-01-25

**Authors:** Kristin Talia Marbun, Marcelia Sugata, Jonathan Suciono Purnomo, Dikson, Samuel Owen Mudana, Tan Tjie Jan, Juandy Jo

**Affiliations:** 1 Department of Biology, Faculty of Science and Technology, Universitas Pelita Harapan, Tangerang 15811, Indonesia; 2 Mochtar Riady Institute for Nanotechnology, Tangerang 15811, Indonesia

**Keywords:** *Bifidobacterium breve*, human breast milk, microbiota, antibiotic resistance, probiotic safety

## Abstract

Our group had isolated *Bifidobacterium breve* strain BS2-PB3 from human breast milk. In this study, we sequenced the whole genome of *B. breve* BS2-PB3, and with a focus on its safety profile, various probiotic characteristics (presence of antibiotic resistance genes, virulence factors, and mobile elements) were then determined through bioinformatic analyses. The antibiotic resistance profile of *B. breve* BS2-PB3 was also evaluated. The whole genome of *B. breve* BS2-PB3 consisted of 2,268,931 base pairs with a G-C content of 58.89% and 2,108 coding regions. The average nucleotide identity and whole-genome phylogenetic analyses supported the classification of *B. breve* BS2-PB3. According to our in silico assessment, *B. breve* BS2-PB3 possesses antioxidant and immunomodulation properties in addition to various genes related to the probiotic properties of heat, cold, and acid stress, bile tolerance, and adhesion. Antibiotic susceptibility was evaluated using the Kirby-Bauer disk-diffusion test, in which the minimum inhibitory concentrations for selected antibiotics were subsequently tested using the Epsilometer test. *B. breve* BS2-PB3 only exhibited selected resistance phenotypes, *i.e.*, to mupirocin (minimum inhibitory concentration/MIC >1,024 μg/ml), sulfamethoxazole (MIC >1,024 μg/ml), and oxacillin (MIC >3 μg/ml). The resistance genes against those antibiotics, *i.e.*, *ileS*, *mupB*, *sul4*, *mecC* and *ramA*, were detected within its genome as well. While no virulence factor was detected, four insertion sequences were identified within the genome but were located away from the identified antibiotic resistance genes. In conclusion, *B. breve* BS2-PB3 demonstrated a sufficient safety profile, making it a promising candidate for further development as a potential functional food.

## Introduction

In recent years, there has been a growing awareness of the potential long-term health benefits associated with the regular consumption of probiotics [1, 2]. Currently, the most common probiotics available on the market contain the bacterial genera *Lactobacillus* and *Bifidobacterium*, which have been discovered to dominate the healthy human gastrointestinal tract (GIT). Such findings led to the utilization of these genera as probiotics in humans [3, 4]. Established studies revealed the ability of these probiotics to modify the host immunological response, compete for adhesion sites with pathogenic microorganisms, and counteract pathogenic microbes [5]. Probiotics, particularly those derived from *Bifidobacterium*, offer a range of health benefits. These include promoting bone health, improving nutrition absorption, reducing fat accumulation, and exhibiting antimicrobial, anticancer, and anti-inflammatory properties [6].

*Bifidobacterium breve* is notable for its antimicrobial properties against human pathogens, non-cytotoxic nature, and immunostimulatory abilities. This probiotic strain is commonly used in children as studies have demonstrated that *B. breve* in the GIT of infants could help reduce diarrhea occurrence, prevent allergic diseases, and protect infants from developing necrotising enterocolitis [6-10]. Building on its success in pediatric applications, there is a growing interest in exploring the potential use of *B. breve* for a wider population [11, 12]. According to previous studies, consumption of *B. breve* in adults is associated with various health benefits, ranging from improvement in memory function of older adults with suspected mild cognitive impairment, enhancement of metabolic function in adults with obese tendencies correlated with reduction of fat mass shown by an increase of gamma-glutamyl transpeptidase (γ-GTP) levels, improvement in diabetic control as shown by the normalization of HbA1c, as well as a significant reduction of severe atopic dermatitis symptoms in adults, hence improving their quality of life [12-14].

Probiotics are usually consumed by incorporating them into food products or directly in the form of dietary supplements. For example, *B. breve* MCC1274 has been used in the form of a freeze-dried powder which is added to food products, such as yogurt, cheese, milk powder products, and even candies [12]. It is crucial, however, to screen the safety of probiotics prior to human consumption [3]. The Food and Agriculture Organization (FAO) of the United Nations and the World Health Organization (WHO) have stated that probiotics should be non-pathogenic and susceptible to clinically relevant antibiotics, while also having the ability to survive simulated GIT conditions, colonize the epithelium, and prevent pathogen adherence to the epithelial cells of GIT [3]. Each regulatory body thus expects a thorough safety evaluation of novel probiotic strains at the biochemical and genetic levels prior to approving their commercial use. The safety evaluation especially highlights the antibiotic resistance profile of probiotics [15]. Although an intrinsic resistance is relatively safe (*e.g.*, *Bifidobacterium*'s natural resistance to mupirocin), non-intrinsic or acquired resistance may be dangerous as it can be transferred to the host's gut microbiota via horizontal gene transfer [16]. Moreover, presence of mobile genetic elements within the genome of probiotics could indicate a transferability of acquired antibiotic resistance gene. Therefore, it is crucial to assess those factors in determining the potential commercial viability of a novel probiotic strain.

We recently isolated a *B. breve* BS2-PB3 from a human breast milk sample in Indonesia and partially sequenced its 16S rRNA gene using Sanger sequencing (manuscript in submission). The isolation from human breast milk indeed highlighted its natural presence in humans. We then aimed to sequence the whole genome of *B. breve* BS2-PB3 and assess its probiotic characteristics, including its safety profile according to FAO-WHO regulations. Furthermore, bioinformatic analyses were performed to confirm the presence of *B. breve* BS2-PB3 and to annotate the genome of *B. breve* BS2-PB3. The safety profile of *B. breve* BS2-PB3 was subsequently evaluated in silico to determine the strain’s antibiotic resistance genes, virulence factors, and mobile elements. Finally, we conducted in vitro measurement of the strain’s antibiotic resistance phenotype via antibiotic disk-diffusion and Epsilometer tests.

## Materials and Methods

### Culture Isolation and Maintenance

Bacterial isolate BS2-PB3 was isolated from human breast milk at the Department of Biology, Universitas Pelita Harapan, and the biochemical and morphological characteristics suggested the strain’s identity as *B. breve*. Molecular identification via the 16S rRNA sequencing using Sanger sequencing was also performed, and indicated the strain to be *B. breve* (manuscript in submission). *B. breve* BS2-PB3 was cultured in Trypticase Phytone Yeast (TPY) medium, containing Tryptic Soy Broth (HiMedia Laboratories, India), peptone (Thermo Fisher Scientific, USA), D-glucose (Sigma-Aldrich, USA), yeast extract (HiMedia Laboratories, India), Tween-80 (Sigma-Aldrich), L-cysteine (NOWFoods, Canada), as well as K_2_HPO_4_ (Sigma-Aldrich). The culture was incubated under anaerobic condition by using Oxoid AnaeroGen 2.5L sachet (Thermo Fisher Scientific) at 37°C for 72 h.

### Whole-Genome Sequencing

The genomic DNA of *B. breve* BS2-PB3 was extracted and sequenced with the Oxford Nanopore Technology (Oxford). Genome assembly was conducted based on EPI2MELABS wf-bacterial-genome pipeline, in which the resulting sequencing reads were assembled de novo with FlyE v2.9.1 (https://github.com/fenderglass/Flye) [17]. The quality control of the final assembly was conducted with Pomoxis v0.3.12 (https://github.com/nanoporetech/pomoxis). The assembled output (FASTA) was annotated with dFAST v1.6.0 (https://dfast.ddbj.nig.ac.jp/) for gene prediction and functional annotation [18]. The circular map of the assembled genome of *B. breve* BS2-PB3 was visualized with Proksee (https://proksee.ca/) [19].

### Bioinformatic Analyses

The whole genome of *B. breve* BS2-PB3 was annotated with dFAST. Various probiotic properties were investigated by screening probiotic-related genes in the dFAST annotated genome. The virulence factors were screened using the virulence factor database (VFDB) (http://www.mgc.ac.cn/VFs/) [20]. The mobile elements were screened using the ISFinder (https://isfinder.biotoul.fr/) [21]. The antibiotic resistance genes were screened using the Comprehensive Antibiotic Resistance Database (CARD) v3.2.7 (https://card.mcmaster.ca/analyze/rgi) [22].

### Antibiotic Resistance Assays

The Kirby-Bauer disk diffusion method was performed based on the Clinical and Laboratory Standards Institute reference method [23]. *B. breve* BS2-PB3 was grown in TPY medium for 36 h and diluted to reach 1.0 turbidity (A_625_). One hundred μl of *B. breve* BS2-PB3 culture was pipetted onto the Mueller-Hinton agar (Condalab, Spain). A sterilized cotton swab was subsequently used to spread the culture evenly over the Mueller-Hinton agar (Himedia, India). Seventeen antibiotic disks (Liofilchem, Italy) were tested, including aminopenicillins (Ampicillin [10 μg]), polypeptide (Bacitracin [10 IU]), cephalosporin (Cefoxitin [30 μg]), penicillinase-resistant penicillin (Oxacillin [1 μg]), glycopeptide (Vancomycin [30 μg]), aminoglycoside (Kanamycin [30 μg] and Streptomycin [10 μg]), tetracycline (Tetracycline [30 μg]), phenicols (Chloramphenicol [30 μg]), lincosamide (Clindamycin [2 μg] and Lincomycin [2 μg]), macrolides (Erythromycin [15 μg]), pleuromutilin (Lefamulin [2 μg]), folate antagonists (Sulfonamide [300 μg]), quinolones (Ciprofloxacin [5 μg]), rifampin (Rifampicin [5 μg]), as well as monocarboxylic acid (Mupirocin [200 μg]). Each antibiotic disk was placed onto the surface of the inoculated agar plate within 30 min after inoculation. The agar plates were incubated in an anaerobic condition at 37°C for 48 h. The measurement of clear zones afterwards included the diameter of antibiotic discs (6 mm). All antibiotic discs were tested three times.

The Epsilometer test (Etest) is a diffusion method, in which a strip impregnated with an increasing concentration gradient of the antibiotic across its length is deposited on an agar plate inoculated with the bacterium of interest [23]. This test provides a result of minimum inhibitory concentration (MIC). Three Etest antibiotic strips were selected based on resistance results of the disk diffusion test, *i.e.*, oxacillin (0.016-256 μg/ml), mupirocin (0.064-1,024 μg/ml), and sulfamethoxazole (0.064-1,024 μg/ml). Each antibiotic strip was placed onto the surface of the inoculated agar plate within 30 min after inoculation. The agar plates were incubated in an anaerobic condition at 37°C for 48 h and inhibition zones were subsequently observed. All antibiotic discs were tested three times.

## Results and Discussion

### Genome Characteristics and Species Confirmation

**Fig. 1:**
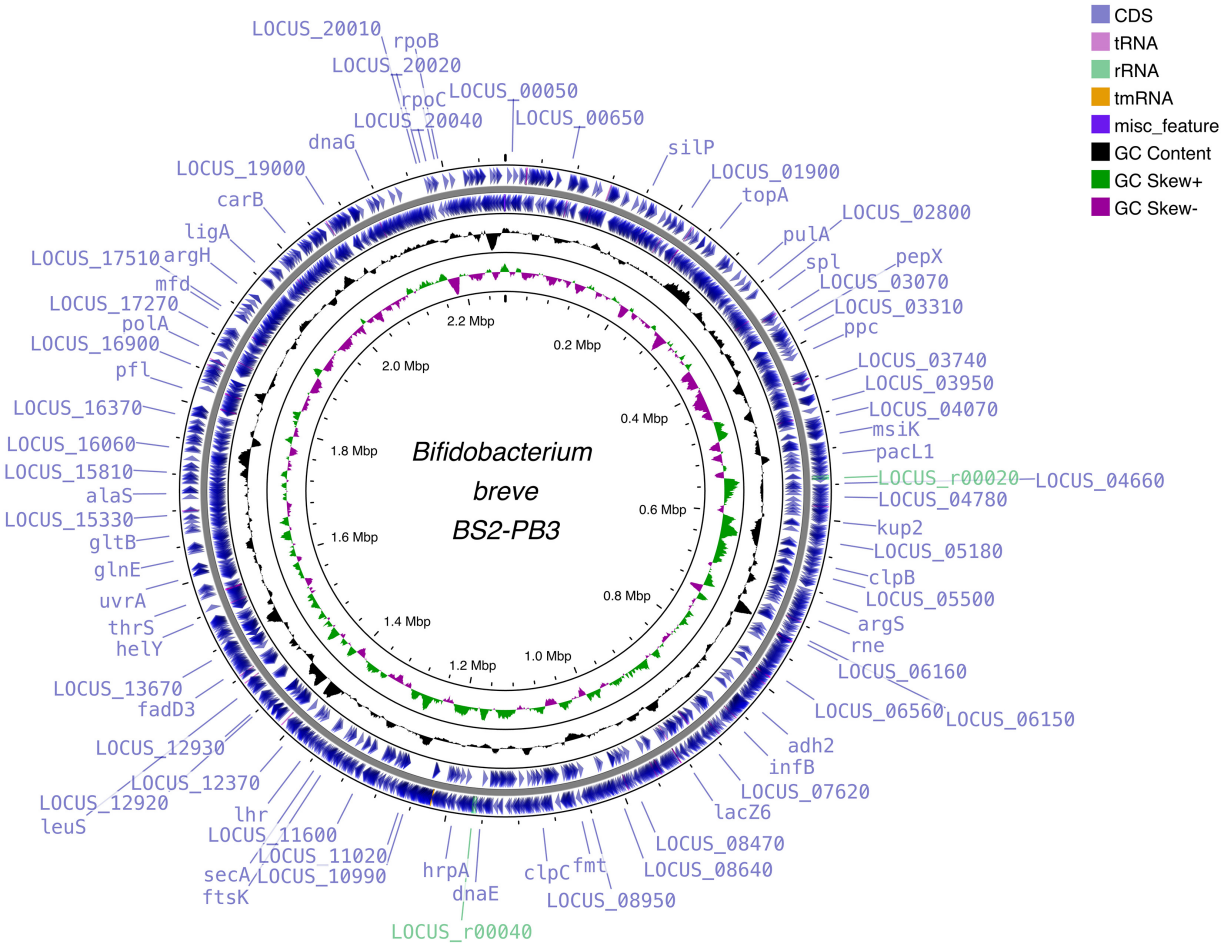
A visualization of whole genome of *Bifidobacterium breve* BS2-PB3. The visualization was performed with Proksee (https://proksee.ca/). Various genes, with hypothetical genes labelled by locus location, were depicted on the outermost violet circle. The first two outermost circles illustrated forward and reverse coding sequences (CDS), with hypothetical genes labelled as the locus location. The CDS was supplemented with tRNAs (pink), rRNAs (light green), and tmRNAs (orange). The third middle circle represented the GC content (black), and the fourth inner circle represented the GC skew (dark green and pink). The fifth innermost circle showed the genome size (*i.e.*, 2,268,931 bp).

**Fig. 2:**
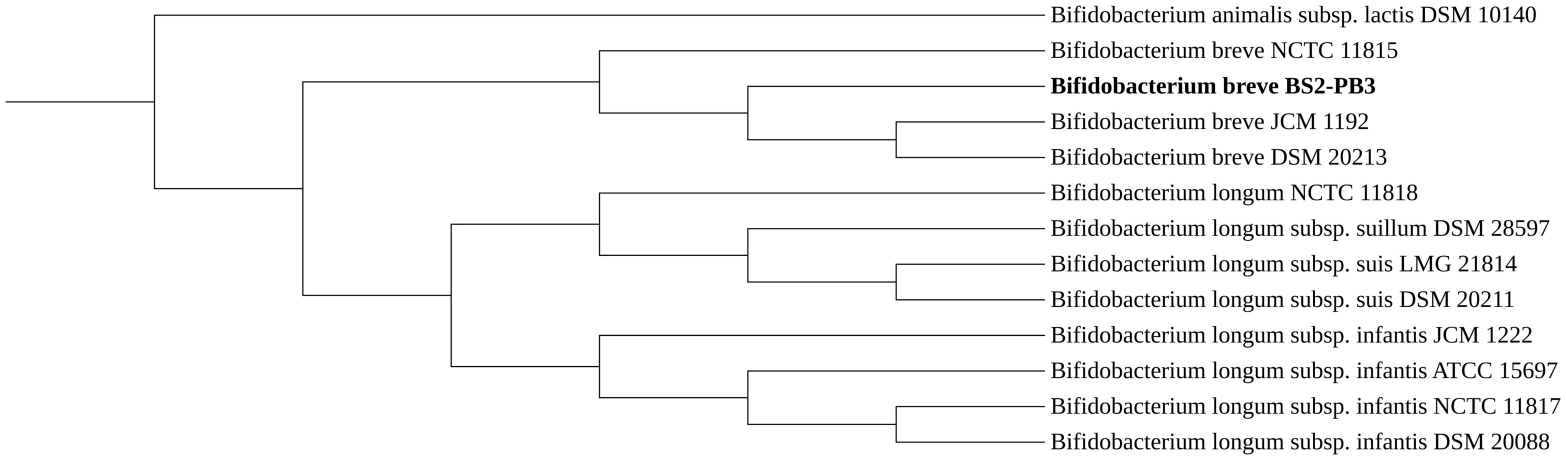
Phylogenetic tree of *Bifidobacterium breve* BS2-PB3. The comparisons of *B. breve* BS2-PB3 with other *Bifidobacterium* strains were carried out in the TYGS webserver (https://tygs.dsmz.de/) and were visualized by iTOL (https://itol.embl.de/). The tree was generated with TYGS, in which the MASH algorithm was used to compare *B. breve* BS2-PB3 with various strains of *Bifidobacterium breve*, *Bifidobacterium animalis* subspecies *lactis*, *Bifidobacterium longum* subspecies *suillum*, *Bifidobacterium longum* subspecies *suis* and *Bifidobacterium longum* subspecies *infantis* in the TYGS database. All pairwise comparisons were conducted using GBDP and accurate intergenomic distances were inferred under the algorithm 'trimming' and distance formula d5. The resulting intergenomic distances were used to infer a balanced minimum evolution tree with branch support via FASTME 2.1.6.1, including SPR postprocessing. Each branch support was inferred from 100 pseudo-bootstrap replicates. The tree was then visualized with iTOL.

The whole genome of *B. breve* BS2-PB3 had a total length of 2,268,931 bp with a G-C content of 58.9%. The Pomoxis tool calculated an error rate of 0.309%, ensuring the quality of its assembly [24, 25]. The complete genome sequence has been submitted to GenBank (Accession No. CP138211). The genome annotation with dFAST predicted a total of 2,133 genes, including 2,108 protein-coding sequences (CDS), 53 tRNAs, 6 rRNAs, and a coding ratio of 86.6% (Table 1). Among predicted CDS, 1,613 proteins were presumed to be functional, while 495 proteins were hypothetical or unknown. Fig. 1 depicts the circular genome map of *B. breve* BS2-PB3. The average nucleotide identity (ANI) and whole-genome phylogenetic analysis were subsequently calculated by the dFAST. The ANI values >95% were often used as a criterion to confirm the species [11]. The ANI values of *B. breve* BS2-PB3 indicated that the strain belongs to *B. breve* with 99% identity. The phylogenomic analysis with TYGS revealed that the *B. breve* BS2-PB3 was closely related to *B. breve* DSM 20213 and JCM 1192 (Fig. 2). In addition, the genome size and G-C content of *B. breve* BS2-PB3 were comparable to those of the other *B. breve* strains [26, 27]. Taken together, these results supported the presence of *B. breve* BS2-PB3.

### In Silico Screening of Probiotic Properties

Probiotic strains possess a large number of genes responsible for maintaining stress response (*e.g.*, against pH and oxidative stress), bile salt hydrolase activity, adhesion ability, and immunomodulatory activities [10, 28]. The screening for those probiotic-associated genes within *B. breve* BS2-PB3 genome were thus conducted by assessing the annotated gene following the criteria established by Kandasamy *et al*. (2022) [1]. Table 2 displays the presence of various probiotic-associated genes within the genome of *B. breve* BS2-PB3. First, the genes associated with heat stress were noted, including heat shock proteins (*htpX*), transcriptional repressors (*hrcA*), and molecular chaperones (*dnaK*, *dnaJ*, *groL*, *groS*, *lon*, *clpB*, *clpC*, *clpX*, *clpP1*, and *clpP2*). The presence of these genes suggested the capability of *B. breve* BS2-PB3 to respond to elevated temperatures [29]. Second, *B. breve* BS2-PB3 possesses the cold shock protein gene (*cspA*), suggesting its ability to adapt to lower temperatures [30]. Third, the genes associated with acid stress (*atpA*, *atpB*, *atpC*, *atpD*, *atpE*, *atpF*, *atpG*, and *atpH*) were detected within the *B. breve* BS2-PB3 genome, indicating a plausibility for the isolate to stay resilient in acidic environments, thus highlighting its resilience in the GIT [30]. Fourth, the genome of *B. breve* BS2-PB3 suggested an ability to tolerate exposure to bile salts due to the presence of the *icfA* gene. This gene encodes carbonic anhydrase, which could sustain the ability of *B. breve* BS2-PB3 to tolerate bile salts in the digestive system [30, 31].

Fifth, the adhesion-related genes (*lspA*, *tuf*, *gpr*, *gap*, *bga*, *eno*, *pgi*, and *fimA*) were also present within the genome of *B. breve* BS2-PB3, indicating the bacterium’s potential to adhere to host cells. This is crucial for probiotics in establishing a beneficial presence in the gut [32]. Sixth, the antioxidant properties of *B. breve* BS2-PB3 were indicated by the presence of genes *fdxC*, *nrdH*, *mntH*, *nox*, *baiC*, and *msrA*. This would permit *B. breve* BS2-PB3 to counteract reactive oxygen species during oxidative stress [33]. Finally, the immunomodulatory capability of *B. breve* BS2-PB3 was suggested by the presence of genes *ddl* and *dacA*. These genes are involved in the synthesis of D-alanine, potentially contributing to the modulation of the host immune response [34]. Collectively, these findings indicate that *B. breve* BS2-PB3 possesses certain genes related to various probiotic properties

### Antibiotic Resistance

*B. breve* BS2-PB3 was screened against 17 antibiotic discs from various classes using the Kirby-Bauer disk-diffusion method on Mueller-Hinton agar. Included among these antibiotic classes were six (aminopenicillins, aminoglycosides, macrolides, lincosamides, tetracyclines and amphenicols) required by the EFSA guidelines (Supplementary Table) [35, 36]. Table 4 summarizes inhibition zone results upon a 2-day incubation, in which *B. breve* BS2-PB3 was interestingly only resistant to mupirocin, sulfonamide, and oxacillin.

Subsequently, the Etest antibiotic strips of mupirocin, sulfamethoxazole and oxacillin were performed to determine the respective MIC of *B. breve* BS2-PB3. Of note, sulfamethoxazole was used as a surrogate of sulfonamide due to the common usage of sulfamethoxazole. Table 5 summarizes the Etest results upon a 2-day incubation, in which *B. breve* BS2-PB3 was indeed resistant to mupirocin (MIC >1,024 μg/ml), sulfamethoxazole (MIC >1,024 μg/ml), and oxacillin (MIC >3 μg/ml).

The resistance towards mupirocin was expected of *B. breve* BS2-PB3 because mupirocin was commonly utilized as a selective agent for isolation of various *Bifidobacterium* spp. from environmental samples, such as intestinal biopsy specimens or human breast milk samples [37, 38]. *Bifidobacterium* spp. are known to be intrinsically resistant to several antibiotics and they are not commonly known to harbor transferrable antibiotic resistance genes, with *tet(W)* being a notable exception [39]. Next, the oxacillin resistance in *B. breve* BS2-PB3 was unexpected because *Bifidobacterium* spp. were supposed to be susceptible to various antibiotics against gram-positive bacteria, including oxacillin [40, 41]. However, some *Bifidobacterium* spp. were reported to possess intrinsic resistance genes to penicillin beta-lactam antibiotics [39]. The resistance of *B. breve* BS2-PB3 towards oxacillin should not be a major issue, arguably, because it was still sensitive to ampicillin, bacitracin, and cefoxitin (Table 4). In the context of sulfonamide resistance, past studies reported that *Bifidobacterium* spp. had an intrinsic resistance to sulfamethoxazole with a reported MIC >128 μg/ml [42-44]. The sul genes had been reported to mediate the sulfonamide resistance in *Bifidobacterium* spp., in which sul3 was the most common variant, presenting in 90.4% of sulfonamide resistance cases [44].

### In Silico Screening of Antibiotic Resistance Genes

Consistent with the results of antibiotic resistance assays, the CARD analysis of the *B. breve* BS2-PB3 genome revealed the presence of resistance genes against oxacillin (*mecC* and *ramA*), sulfonamide (*sul4*), and mupirocin (*ileS* and *mupB*) [22]. The *mecC* gene, a homolog of *mecA* gene, encoded an alternative penicillin-binding protein (PBP2a or PBP2c) that possessed a reduced affinity for beta-lactam antibiotics [45-47]. The *ramA* gene encoded a transcriptional regulator that influences the expression of multiple efflux pumps that was also detected during the CARD analysis [48]. Next, a sulfonamide resistance gene, *sul4*, encoded a modified sulfonamide-resistant dihydropteroate synthase (DHPS), a key enzyme in folate biosynthesis [49, 50]. The modified DHPS exhibited a decreased affinity for sulfamethoxazole, impacting the effectiveness of sulfonamide drugs to target the folate biosynthesis pathway [49, 50].

*B. breve* BS2-PB3 was categorized as having high-level mupirocin resistance because its MIC of mupirocin was ≥512 μg/ml [51, 52]. This strain exhibited both low- and high-level genetic resistance to mupirocin. The low-level resistance was attributed to a mutation within the *ileS* gene, a gene encoding isoleucyl-tRNA synthetase. This enzyme plays a crucial role in protein synthesis by ensuring the accurate incorporation of isoleucine into growing polypeptide chains. The mutation resulted in amino acid substitutions at V588F and V631F within the ATP-binding domain of the Rossman fold. These substitutions occurred in a region that overlaps with the mupirocin-binding domain [53, 54], affecting the functionality of the IleS enzyme. The modified IleS enzyme had a reduced affinity for mupirocin, resulting in decreased binding of the antibiotic to its target site. High-level mupirocin resistance was contributed by the presence of *mupB* gene. *mupB* regulates the production of the target enzyme, allowing bacteria to increase their levels in response to the antibiotic [52, 55]. *B. breve* BS2-PB3 was observed to possess *mupB* through genome annotation analysis and sequence alignment with *mupB* from GenBank. *B. breve* BS2-PB3 possessed the conserved motifs typically observed in class I tRNA synthetases, which play a crucial role in ATP hydrolysis (HTGH and KMSKS). *B. breve* BS2-PB3 also possessed the GWD motif, commonly conserved in isoleucyl-tRNA synthetases, that was responsible for amino acid activation [52].

### In Silico Screening of Virulence Factors and Insertion Sequence

The virulence factor database (VFDB) was used to identify virulence factors that might exist within the genome of *B. breve* BS2-PB3 [20]. No virulence gene was found either under the stringent criteria (80% identity and >60%coverage with E-value <1 × 10^-10^) or the less stringent criteria (>60% identity and >60% coverage with E-value <1 × 10^-10^). Next, mobile elements within the genome of *B. breve* BS2-PB3 were assessed with the ISFinder [21]. A total of 34 insertion sequence (IS) elements belonging to five families (IS256, IS3, IS607, IS21, and IS30) were identified in the genome with the set threshold E-value of 0.0001 (Table 3). Four insertion sequences (ISBibr1, ISBIo9, ISBIo12, and ISBIo5) were significantly matched with the query sequence (E-value of 0), suggesting that these IS elements were likely present in the genome of *B. breve* BS2-PB3. Such as result could have negative consequences because IS elements are DNA segments that can relocate within a genome and play a role in promoting genetic diversity in bacteria [45]. Nonetheless, these insertion sequences were not located near the identified antibiotic resistance genes (*mecC*, *ramA*, *sul4*, *ileS*, and *mupB*) within the *B. breve* BS2-PB3 genome (Table 6). Therefore, the absence of nearby insertion sequences, arguably, would reduce the risk of the horizontal gene transfer of those antibiotic resistance genes to other bacteria. Taken together, these findings supported the safety profile of *B. breve* BS2-PB3.

## Conclusion

The whole genome of *B. breve* BS2-PB3, isolated from a human breast milk sample in Indonesia, has a total length of 2,268,931 bp with 2,133 genes. The in silico investigation suggested that this strain contains various genes related to probiotic properties, including resistance to heat stress, cold shock, acid stress and bile salts, as well as having abilities associated with adhesion, antioxidation, and immunomodulation. *B. breve* BS2-PB3 demonstrated resistance phenotypes only to mupirocin, sulfonamide, and oxacillin, in which these phenotypes were corroborated by the presence of relevant antibiotic resistance genes (*ileS*, *mupB*, *sul4*, *mecC*, and *ramA*). *B. breve* BS2-PB3 was not observed to possess any virulence genes. In conclusion, our findings demonstrated that *B. breve* BS2-PB3 possesses a safety profile that makes this strain a promising candidate for further development as a potential functional food.

## Supplemental Materials

Supplementary data for this paper are available on-line only at http://jmb.or.kr.



## Figures and Tables

**Table 1:** Genome annotation statistics of *Bifidobacterium breve* BS2-PB3 using the dFAST annotation service.

Attribute	*B. breve* BS2-PB3
Genome size (bp)	2,268,931
Contig	1
GC content (%)	58.89
Contig N50 (bp)	2,268,931
Contig L50	1
Plasmids	0
CDS	2,108
Total RNA's	58 (54 tRNA + 4 rRNA)
Protein coding sequence	2,108
Number of CRISPRs	0

N50 was related to the median and mean lengths of a set of sequences, in which it represented the length of the shortest read in the group of longest sequences, which together account for at least 50% of the nucleotides in the set of sequences. L50 was related to N50, indicating the number of sequences that, when arranged from longest to shortest, are needed to reach or exceed 50% of the total assembly size. bp, base pairs; GC, guanine-cytosine; CDS, coding sequence; RNA, ribonucleic acid; CRISPR, clustered regularly interspaced short palindromic repeats.

**Table 2:** List of probiotic-associated genes present in the *Bifidobacterium breve* BS2-PB3 genome.

Gene	Function	Locus
Heat stress		
*htpX*	Heat shock protein htpX	LOCUS_02980
*hrcA*	Heat-inducible transcriptional repressor	LOCUS_14510
*dnaK*	HSPA9; molecular chaperone DnaK	LOCUS_02690
*dnaJ*	Molecular chaperone DnaJ	LOCUS_14500, LOCUS_02660
*groL*	HSPD1; chaperonin	LOCUS_09360
*groS*	HSPE1: chaperonin	LOCUS_06630
*lon*	Lon protease	LOCUS_18240
*clpB*	ATP-dependent chaperone protein ClpB	LOCUS_05410
*clpC*	ATP-dependent chaperone protein ClpC	LOCUS_09450
*clpX*	ATP-dependent chaperone protein ClpX	LOCUS_16550
*clpP1*	ATP-dependent chaperone protein ClpP1	LOCUS_16560
*clpP2*	ATP-dependent chaperone protein ClpP2	LOCUS_16570

Cold stress		
*cspA*	Cold shock protein	LOCUS_09350

Acid stress		
*atpA*	F-type H^+^/Na^+^-transporting ATPase subunit alpha	LOCUS_01040
*atpB*	F-type H^+^ transporting ATPase subunit a	LOCUS_01080
*atpC*	F-type H^+^ transporting ATPase subunit epsilon	LOCUS_01010
*atpD*	F-type H^+^/Na^+^-transporting ATPase subunit beta	LOCUS_01020
*atpE*	F-type H^+^ transporting ATPase subunit c	LOCUS_01070
*atpF*	F-type H^+^ transporting ATPase subunit b	LOCUS_01060
*atpG*	F-type H^+^ transporting ATPase subunit gamma	LOCUS_01030
*atpH*	F-type H^+^ transporting ATPase subunit delta	LOCUS_01050

Bile tolerance		
*icfA*	Carbonic anhydrase	LOCUS_03630

Adhesion		
*lspA*	Lipoprotein signal peptidase II	LOCUS_10000
*tuf*	Elongation factor Tu	LOCUS_18900
*gpr*	L-glyceraldehyde 3-phosphate-reductase	LOCUS_00250
*gap*	Type 1 glyceraldehyde 3-phosphate-reductase	LOCUS_11410
*bga*	Beta galactosidase	LOCUS_19770
*eno*	Enolase	LOCUS_17460
*pgi*	Glucose-6-phosphate isomerase	LOCUS_00180
*fimA*	Type-1 fimbrial protein	LOCUS_02790

Antioxidant		
*fdxC*	Ferredoxin	LOCUS_06680
*nrdH*	Glutaredoxin	LOCUS_04130
*mntH*	manganese transport protein	LOCUS_20690
*nox*	NADH oxidase	LOCUS_05700
*baiC*	NADH-dependent flavin oxidoreductase	LOCUS_20190
*msrA*	Peptide-methionine (S)-S-oxide reductase	LOCUS_03110

Immunomodulation		
*ddl*	D-alanine-D-alanine ligase	LOCUS_00900
*dacA*	D-alanyl-D-alanine carboxypeptidase	LOCUS_08060

Probiotic-associated genes were screened manually by screening the probiotic properties-related genes [1].

**Table 3:** List of insertion sequences present in the *Bifidobacterium breve* BS2-PB3 genome using the ISFinder.

Insertion Sequence	IS Family	Group	Origin	Location in the genome (bp)	E value
ISBlbr1	IS256	IS1249	*Bifidobacterium breve*	173.720-175.339	00.00
ISBlo9	IS3	IS150	*Bifidobacterium longum*	1.428.521-1.429.711	00.00
ISBlo12	IS607	-	*Bifidobacterium longum*	1.433.778-1.434.968	00.00
ISBlo5	IS256	-	*Bifidobacterium longum*	1.837.125-1.838.135	00.00

bp, base pair; IS, insertion sequence.

**Table 4:** Antibiotic resistance profile of *Bifidobacterium breve* BS2-PB3 based on the disk-diffusion method.

Antibiotic class	Antibiotic	Clear zone diameter (mm)	R/S/I
Aminopenicillin	Ampicillin (10 µg) ^a^	49 ± 0.60	S
Bacitracin	Bacitracin (10 IU) ^a^	58 ± 2.31	S
Glycopeptide	Vancomycin (30 µg)^a^	48 ± 2.65	S
Cephalosporine	Cefoxitin (30 µg) ^a^	26 ± 3.21	S
β-Lactams	Oxacillin (1 µg)^b^	6 ± 0.00	R
Amphenicols	Chloramphenicol (30 µg)^a^	63 ± 2.08	S
Macrolides	Erythromycin (15 µg)^a^	62 ± 1.53	S
	Streptomycin (10 µg)^a^	37 ± 2.52	S
Aminoglycosides	Kanamycin (30 µg)^a^	20 ± 4.00	S
Tetracycline	Tetracycline (30 µg)^a^	57 ± 2.65	S
	Clindamycin (2 µg)^b^	56 ± 5.51	S
Lincosamides	Lincomycin (2 µg)^c^	50 ± 3.61	S
Pleuromutilins	Lefamulin (20 µg) ^e^	75 ± 2.08	S
Monocarboxylic acid	Mupirocin (200 µg)^d^	11 ± 1.15	R
Quinolones	Ciprofloxacin (5 µg) ^a^	32 ± 2.89	S
Rifampicin	Rifampicin (5 µg)^a^	64 ± 1.15	S
Sulfonamide	Sulfonamide (300 µg)^a^	7 ± 1.15	R

Diameter of clear zone/inhibition zone was presented as the mean of three experiments ± SD. ^a^Inhibition zone was based on [58]. ^b^Inhibition zones of oxacillin and clindamycin were based on CLSI standard described by [59]. ^c^Inhibition zone of lincomycin was based on [60]. ^d^Inhibition zone of mupirocin was based on [61]. ^e^Inhibition zone of lefamulin was based on interpretative criteria results of *Staphylococcus aureus* (methicillin-susceptible isolates) as described by [62]. R, Resistance; S: Sensitive; I, Intermediate.

**Table 5:** Minimum inhibitory concentration of *Bifidobacterium breve* BS2-PB3 based on the Epsilometer test.

Antibiotic class	Antibiotic	Minimum Inhibitory Concentration (µg/mL)
β-Lactams	Oxacillin ^a^	3.25 ± 1.89
Monocarboxylic acid	Mupirocin ^b^	>1,024 ± 0.00
Sulfonamide	Sulfamethoxazole ^a^	>1,024 ± 0.00

Diameter of clear zone was presented as the mean of three experiments ± SD. ^a^Minimum inhibitory concentration standard was based on [63]. ^b^Minimum inhibitory concentration standard was based [64].

**Table 6:** Locations of the insertion sequence and identified antibiotic resistance gene of *Bifidobacterium breve* BS2-PB3.

Insertion Sequence	Location in the genome (bp)	Antibiotic Resistance Gene	Location in BS2-PB3 genome (bp)
ISBlbr1	173,720-175,339	*ileS*	1,029,489-1,032,288
ISBlo9	1,428,521-1,429,711	*mupB*	130,301- 133,036
ISBlo12	1,433,778-1,434,968	*mecC*	383,560- 384,177
ISBlo5	1,837,125-1,838,135	*ramA*	1,771,307- 1,772,167
		*sul4*	943,671- 944,549

This table presents a comparison between the positions of insertion sequences and the locations of expressed antibiotic resistance genes within the genome of *B. breve* BS2-PB3. The positions of insertion sequences were identified using the ISFinder (https://isfinder.biotoul.fr/), while the locations of antibiotic resistance genes were determined through BLAST alignment analysis (https://blast.ncbi.nlm.nih.gov/Blast.cgi) of the expressed genome against reference antibiotic resistance genes. bp, base pair; IS, insertion sequence.
