# Supplementary material for: Genomic Characterization and Safety Assessment of Bifidobacterium breve BS2-PB3 as Functional Food
Source: J Microbiol Biotechnol. 2024 Jan 25;34(4):871–9. doi: 10.4014/jmb.2311.11031 (PMC11091697; doi:10.4014/jmb.2311.11031)
Supplement: Supplementary file 1 [file jmb-34-4-871-supple.pdf]

J. Microbiol. Biotechnol.
https://doi.org/10.4014/jmb.2311.11031

**Supplementary Table.** Microbiological cut-off values of *Bifidobacterium* species.

| Class | Antibiotic | MIC cut-off (µg/mL) |
|---|---|---|
| **Aminopenicillin** | Ampicillin | 2 |
| **Glycopeptide** | Vancomycin | 2 |
| **Aminoglycosides** | Gentamycin | 64 |
| | Streptomycin | 128 |
| **Macrolides** | Erythromycin | 1 |
| **Lincosamides** | Clindamycin | 1 |
| **Tetracycline** | Tetracycline | 8 |
| **Amphenicol** | Chloramphenicol | 4 |

Note: Minimum inhibitory concentration (MIC) of antibiotics was based on the EFSA guidelines [37, 38].
